# A systematic review of progress on hepatocellular carcinoma research over the past 30 years: a machine-learning-based bibliometric analysis

**DOI:** 10.3389/fonc.2023.1227991

**Published:** 2023-08-17

**Authors:** Kiseong Lee, Ji Woong Hwang, Hee Ju Sohn, Sanggyun Suh, Sun-Whe Kim

**Affiliations:** ^1^ Humanities Research Institute, Chung-Ang University, Seoul, Republic of Korea; ^2^ Department of Surgery, Chung-Ang University Gwangmyeong Hospital, Chung-Ang University College of Medicine, Gwangmyeong, Republic of Korea

**Keywords:** hepatocellular carcinoma, bibliometric analysis, machine learning, latent Dirichlet allocation, research trend

## Abstract

**Introduction:**

Research on hepatocellular carcinoma (HCC) has grown significantly, and researchers cannot access the vast amount of literature. This study aimed to explore the research progress in studying HCC over the past 30 years using a machine learning-based bibliometric analysis and to suggest future research directions.

**Methods:**

Comprehensive research was conducted between 1991 and 2020 in the public version of the PubMed database using the MeSH term “hepatocellular carcinoma.” The complete records of the collected results were downloaded in Extensible Markup Language format, and the metadata of each publication, such as the publication year, the type of research, the corresponding author’s country, the title, the abstract, and the MeSH terms, were analyzed. We adopted a latent Dirichlet allocation topic modeling method on the Python platform to analyze the research topics of the scientific publications.

**Results:**

In the last 30 years, there has been significant and constant growth in the annual publications about HCC (annual percentage growth rate: 7.34%). Overall, 62,856 articles related to HCC from the past 30 years were searched and finally included in this study. Among the diagnosis-related terms, “Liver Cirrhosis” was the most studied. However, in the 2010s, “Biomarkers, Tumor” began to outpace “Liver Cirrhosis.” Regarding the treatment-related MeSH terms, “Hepatectomy” was the most studied; however, recent studies related to “Antineoplastic Agents” showed a tendency to supersede hepatectomy. Regarding basic research, the study of “Cell Lines, Tumors,’’ appeared after 2000 and has been the most studied among these terms.

**Conclusion:**

This was the first machine learning-based bibliometric study to analyze more than 60,000 publications about HCC over the past 30 years. Despite significant efforts in analyzing the literature on basic research, its connection with the clinical field is still lacking. Therefore, more efforts are needed to convert and apply basic research results to clinical treatment. Additionally, it was found that microRNAs have potential as diagnostic and therapeutic targets for HCC.

## 1 Introduction

According to the World Health Organization, hepatocellular carcinoma (HCC) is the fifth most common neoplasm and the third leading cause of cancer-related death worldwide (1). The major risk factors of HCC include chronic hepatitis B or C virus, excessive alcohol consumption, diabetes, and potentially nonalcoholic fatty liver disease (2). Additionally, liver cirrhosis, regardless of its etiology, is a potential risk factor for the development of HCC (3).

Several recommendations and guidelines have suggested the use of contrast-enhanced ultrasound (CEUS), contrast-enhanced computed tomography (CT), or magnetic resonance imaging (MRI) as the diagnostic modalities for HCC surveillance. Abdominal ultrasound has traditionally been considered the fundamental method for HCC surveillance and is still advocated as the primary test for this purpose. The addition of serum biomarkers, particularly alpha-fetoprotein (AFP), can enhance the sensitivity of abdominal ultrasound in detecting HCC at an early stage (4). Patients with HCC have access to various therapeutic approaches, including liver transplantation, surgical resection, percutaneous ablation, radiation, and transarterial and systemic therapies (5). Therefore, a multidisciplinary approach is necessary to tailor the treatment strategy to the patient’s tumor stage, liver function, and performance status.

Studies on HCC have grown significantly, with researchers being unable to access the vast amount of literature. Thus, to reflect current research trends, bibliometric analysis may be used to collect and investigate the most relevant information from a large volume of literature (6). Furthermore, it can enable the research to progress to a macroscopic perspective and provide directions for future study (7).

Natural language processing (NLP) is a type of machine learning method used for human language analyses. Among these methods, the latent Dirichlet allocation (LDA) is one of the most classical topic modeling techniques and is usually used to analyze scientific publications comprehensively by identifying research topics and categorizing articles into these topics (8, 9). Recently, LDA has been used in several bibliometric analyses in the field of anal cancer, rectal cancer, and cardiovascular research (10–12).

Therefore, this study aimed to explore the research progress in studying HCC over the past 30 years using a machine learning-based bibliometric analysis and to suggest future research directions.

## 2 Methods

In April 2022, comprehensive research was performed in the public version of the PubMed database, using the MeSH term “hepatocellular carcinoma” for the period between 1991 and 2020 (**Figure S1**). The articles that lacked an abstract or were not written in English were excluded. The complete records of the results were downloaded in Extensible Markup Language format, and R was used to extract the metadata of each article, such as the publication year, the type of research, the title, the abstract, and the MeSH terms. Overall, 12,898 MeSH terms were identified in the included publications, and only MeSH terms that appeared 100 times or more were included in this analysis (n = 980). No ethical approval from an institutional review board was required since this study was a bibliometric analysis.

An LDA algorithm was adopted to identify the relevant topics from the abstract of each publication in the Python platform to analyze the research topics in numerous scientific publications. An LDA is an analytical method that identifies the main topic of a document based on the probability of a combination of words that appear together frequently. An LDA generates a topic probability distribution of each document from a large collection of documents, and one topic with the largest weight in the probability distribution becomes the main topic of the document. This study examined the abstract of papers related to HCC published over the past 30 years and calculated the probability of the appearance of each paper’s main topics and vocabulary. The number of topics was designated as 50 to align with existing research, and the name of the topic was assigned manually based on the vocabulary combination constituting each topic and the included literature title. Furthermore, LDA processing was performed using coding in Python with the gensim package (https://radimrehurek.com/gensim/). A Louvain algorithm was generated to further investigate the relationship between these topics. The network visualizations were constructed using Gephi software (https://gephi.org/) (13). All mentioned codes, including the Python code, were available on Github (https://github.com/kisleepublic/analysisHCC/; GitHub is a website and cloud-based service that helps developers store and manage their code).

## 3 Results

### 3.1 Overview of the data

**Figure 1:**
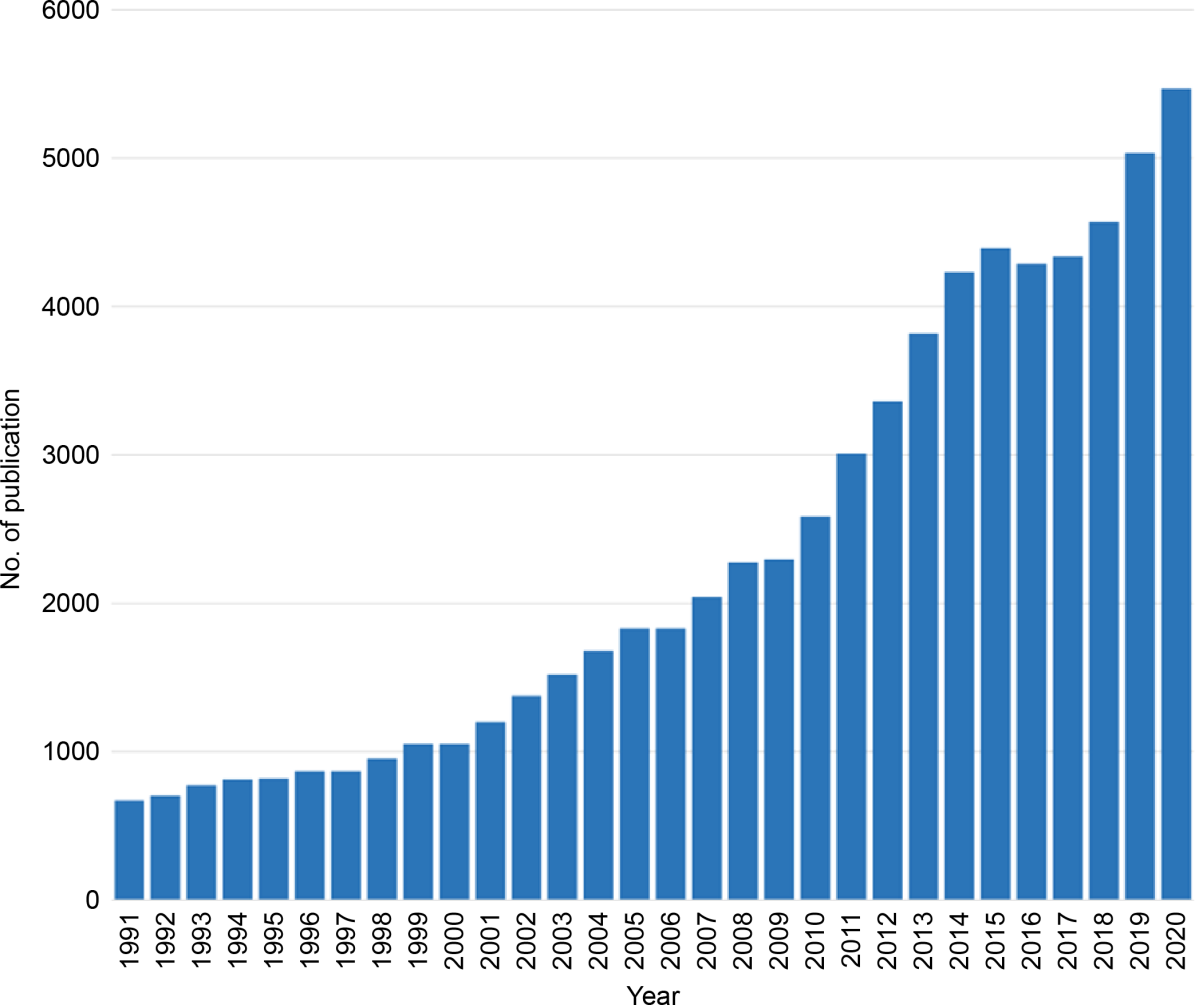
Annual publication on hepatocellular carcinoma.

Overall, 62,856 articles related to HCC over the past 30 years were searched and finally included in this bibliometric analysis. We found that 671 publications on HCC research were available in 1991, which increased to 5,240 in 2020 (**Figure 1**). In the last 30 years, there has been significant and constant growth in the annual publications about HCC (annual percentage growth rate: 7.34%).

**Figure 2:**
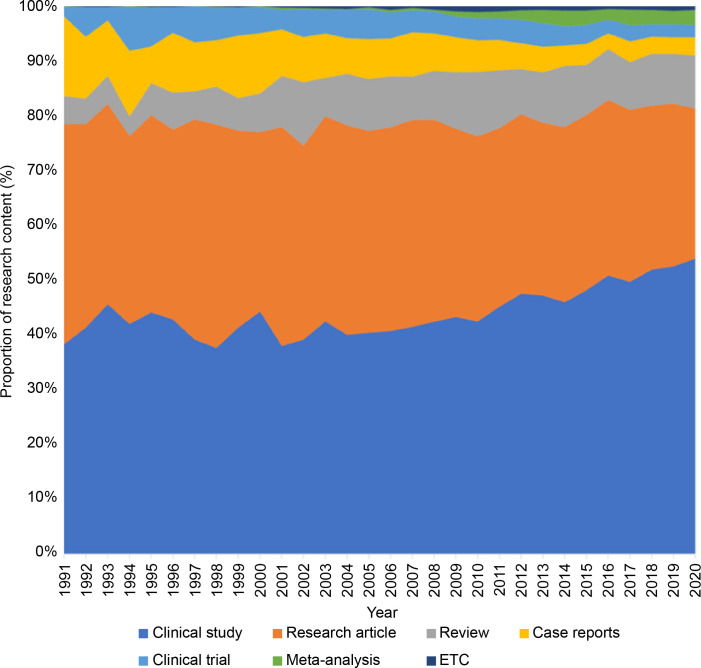
Distribution of publication types per year.

The highest number of publications involved clinical studies (n = 30,115, 47.9%), followed by research articles (n = 21,530, 34.3%) and reviews (n = 5,851, 9.3%). **Figure 2** shows the distribution of the publication types by year. Clinical studies accounted for approximately half of the publications, and their proportion increased steadily. The proportion of research articles, case reports, and clinical trials decreased, whereas that of reviews and meta-analyses tended to increase gradually.

**Figure 3:**
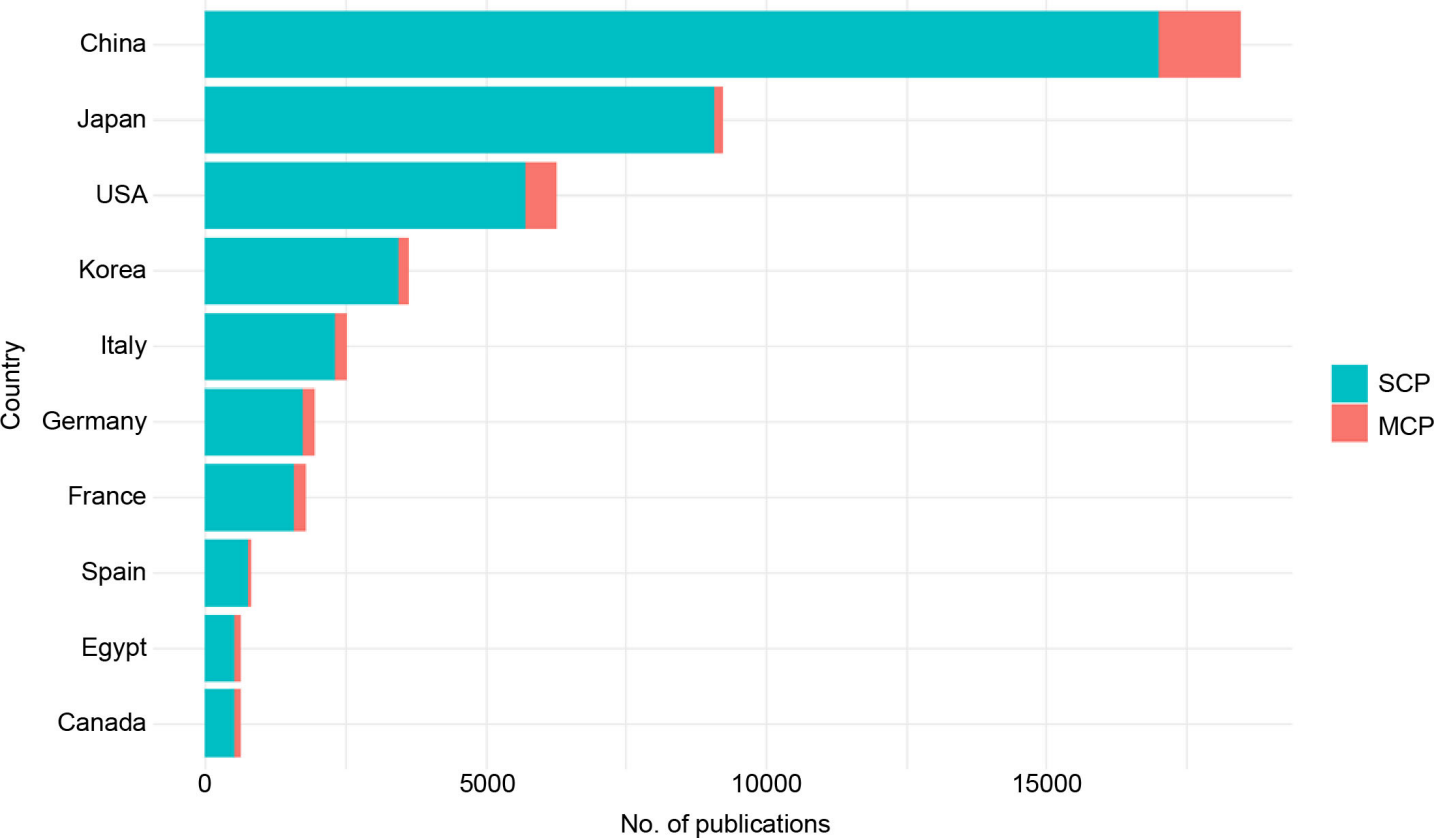
The contribution of the country to publication on hepatocellular carcinoma. SCP, single country publications; MCP, multiple country publications.

The most productive country was China (n = 18,476, 35.1%), followed by Japan (n = 9,248, 17.6%) and the United States (n = 6,283, 11.9%) (**Figure 3**). China also had the highest number of multiple country publications (MCP) (n = 1,478, MCP ratio; 0.081), while Egypt had the highest MCP ratio (n = 647, MCP ratio = 0.198) (**Table S1**).

### 3.2 MeSH term analysis

Further analyses were performed using the MeSH terms found in the publications. Notably, 980 MeSH terms appeared 100 times or more and were included in this analysis, with a total of 945,754 times of occurrence (**Supplemental Data 1**). **Table S2** shows the most widely studied top 20 MeSH terms in HCC research. Subsequently, we investigated focused diagnosis-, treatment-, and basic research-related MeSH terms.

**Figure 4:**
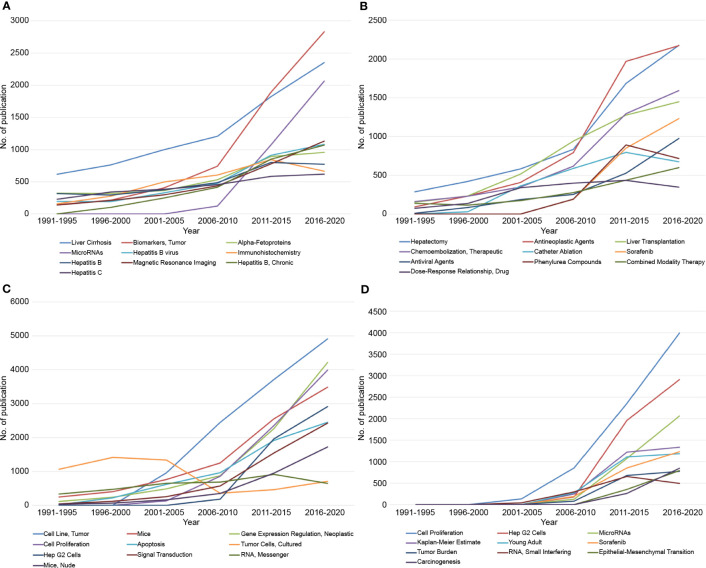
Accumulative occurrence of the top 10 MeSH terms regarding diagnosis **(A)**, treatment **(B)**, basic research **(C)** of hepatocellular carcinoma over a 5-year trend, and newly appeared after 2000 **(D)**.

Among the diagnosis-related terms, “Liver Cirrhosis” was the most studied. However, in the 2010s, “Biomarkers, Tumor” began to outpace “Liver Cirrhosis” (**Figure 4A**). In contrast to the rapid increase of “Biomarkers, Tumors,” the appearance of “alpha-fetoproteins,” considered an important biomarker of HCC, showed a slight increase. Rather, the rapid increase of “Biomarkers, Tumor” was accompanied by an increase of “MicroRNAs.” Regarding the MeSH terms related to hepatitis, which are the representative risk factors of HCC, four terms (“Hepatitis B virus,” “Hepatitis B,” “Hepatitis B, Chronic,” and “Hepatitis C”) were included in the top 10 terms and showed a steadily increasing tendency. Notably, the MeSH terms related to hepatitis B showed a steep increase, whereas “Hepatitis C” demonstrated no significant increase. Regarding imaging modalities, “Magnetic Resonance Imaging” was included in the top 10 terms and showed a steady increase.

Concerning the treatment-related MeSH terms, “Hepatectomy” was the most studied; however, recent studies related to “Antineoplastic Agents” showed a tendency to supersede hepatectomy (**Figure 4B**). The recent increase in research on “Antineoplastic Agents” appears to reflect the increase in studies on “Sorafenib,” which suggested that the increase in research on “Sorafenib” played an important role in the increase in research on “Antineoplastic Agents.” Studies related to other treatment modalities, such as hepatectomy, liver transplantation, and TACE, showed a steady increase, whereas recently, those on “Catheter Ablation” demonstrated a tendency to decrease slightly.

Regarding basic research, the study of “Cell Lines, Tumors,’’ appeared after 2000 and has been the most studied among these terms (**Figure 4C**). Conversely, “Tumor Cells, Cultured’’ were the most studied until the early 2000s; however, there has been a significant decline since then. Among the tumor cell lines, “Hep G2 Cells” were included in the top 10 MeSH terms and appeared in the late 2000s, showing a rapid increase.

By analyzing the MeSH terms that appeared after 2000, a subgroup analysis was also performed on newly emerging research directions. It accounted for 11.6% of the total number of MeSH terms and appeared only 55,988 times (**Figure 4D**). Among these terms, the number of publications with the topics, including “Cell Proliferation,” “Hep G2 cells,” and “microRNAs” substantially increased and appeared in 2003, 2006, and 2006, respectively. Although MeSH terms related to basic research comprised most of the top 10, “Young Adults” and “Sorafenib” were included in the top 10 as clinical-related MeSH terms.

### 3.3 LDA analysis

**Figure 5:**
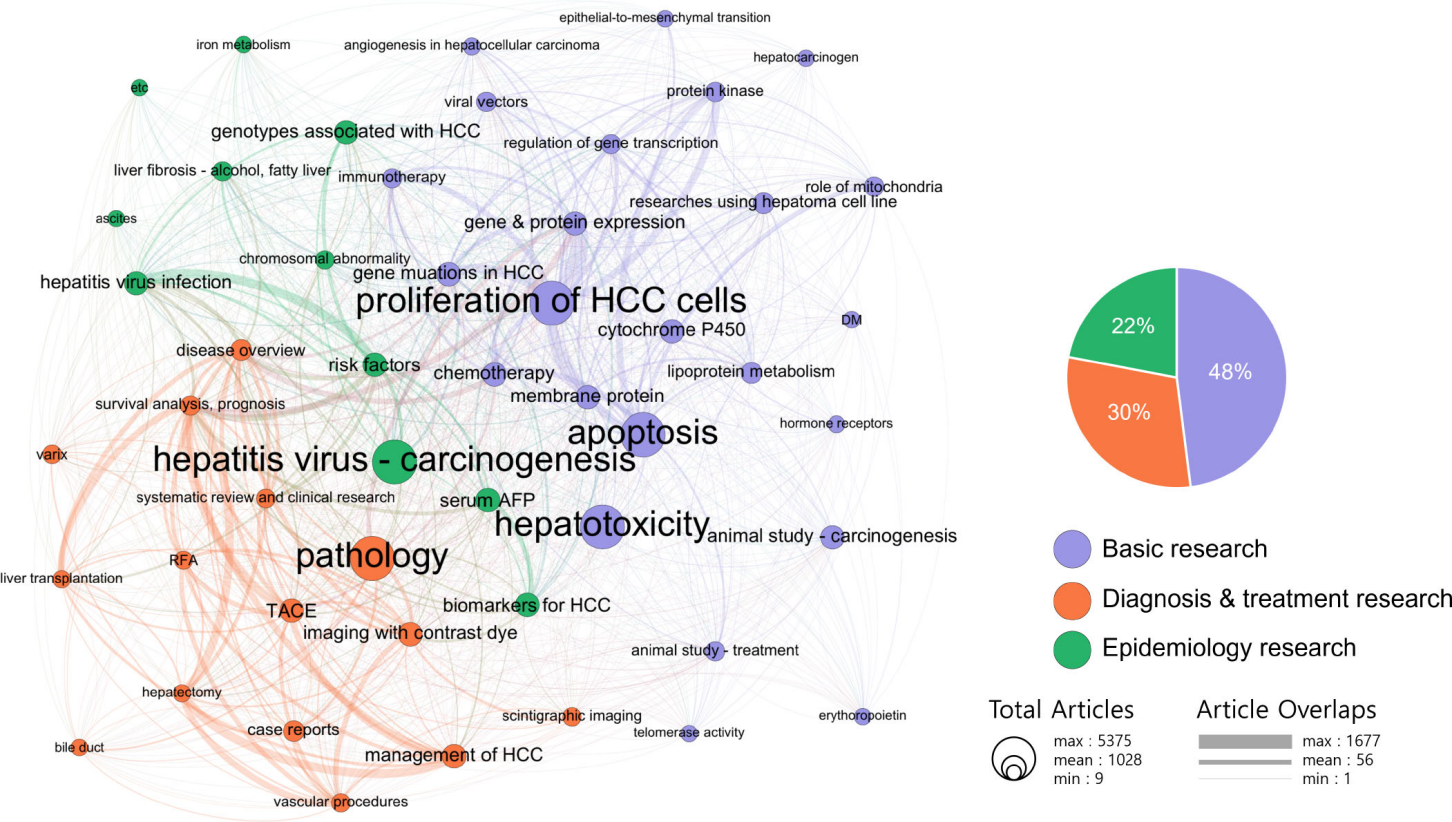
Topic cluster network analysis using latent Dirichlet allocation. Purple, basic research; orange, diagnosis and treatment research; and green, epidemiology research. The size of the circle represents the number of articles on each topic, and the thickness of the line represents the weight of the connection between each topic.

Overall, 61,891 publications with available abstracts were analyzed using the LDA method, excluding those without an abstract. The LDA method provided further information about popular topics and their relationships by generating a topic network from the abstract text analysis (**Figure 5**). The network was categorized into three clusters, including “basic research” (in purple), “diagnosis and treatment research” (in orange), and “epidemiology research” (in green). The focalization of topics and the weight of connections between the topics were demonstrated as the size of the circle and the thickness of the line, respectively.

The cluster of basic research constituted the largest proportion of these topics, where “proliferation of HCC cells,” “apoptosis,” and “gene and protein expression” were the top three research topics. In the cluster of diagnosis and treatment research, “survival analysis, prognosis,” “imaging with contrast dye,” and “TACE” were the top three research topics. In the cluster of epidemiology research, “risk factor,” “hepatitis virus infection,” and “hepatitis virus_carcinogenesis” were the top three research topics.

The basic research cluster included half of the entire set of publications but had a poor connection with the other two clusters, while comparatively, the epidemiology research cluster showed many associations with other clusters. Except for “gene and protein expression,” most of the topics in the basic research cluster had a strong connection only to other topics in the basic research cluster. The topics “gene and protein expression” demonstrated strong connections to the topics “survival analysis, prognosis” and “pathology” in other clusters.

## 4 Discussion

To date, the analysis of publications, which grew in volume over time, has been conducted directly by researchers, resulting in a limit to the number of publications that could be analyzed. With the development of artificial intelligence technology, the bibliographic analysis of numerous publications has become possible. A bibliometric analysis is usually performed to analyze research trends in various academic fields and to help suggest future research directions. We used an NLP, which is a type of machine learning to systematically analyze 62,856 HCC-related literature published over the past 30 years. To the best of our knowledge, this was the first machine learning bibliometric analysis on HCC research. We also visualized the results from a macroscopic perspective to make the analysis of the detected trends more intuitive.

The AFP is currently the most accepted phase 5 biomarker and is used frequently in the surveillance and diagnosis of HCC (14). However, AFP has limitations as a biomarker due to its low sensitivity and specificity for early HCC (15). As shown in **Figure 4A**, the topic “Biomarkers Tumor” has grown significantly, while the number of publications on the topic “alpha-fetoproteins” has increased slightly, indicating that research on novel biomarkers for HCC, particularly for early HCC, is needed, and that many related studies have been conducted. **Figure 4D** shows that “MicroRNAs” appeared after 2000 with a rapidly increasing frequency. The recent rapid increase in “Biomarkers, Tumor” may be due to the increase in these “MicroRNAs.” MicroRNAs play an important role in the pathogenesis of HCC and are expressed differentially even in the early stages of HCC (16). The emerging role of microRNAs as novel potential biomarkers *via* early diagnosis may change the face of HCC surveillance (17, 18).

The European Association for the Study of the Liver guidelines has recommended the use of CEUS, contrast-enhanced CT, or MRI as the diagnostic modalities for HCC surveillance (19). Many studies have suggested that MRI has a higher diagnostic value than CEUS or CT, particularly for small HCC, because of its higher sensitivity and accuracy (20, 21). Our study also demonstrated that “MRI” was the only imaging modality included in the top 10 diagnosis-related MeSH terms, and its frequency of appearance incidence has increased steadily.

Generally, therapeutic strategies for patients with HCC are determined based on Barcelona Clinic Liver Cancer criteria (2). In the early stage, surgical resection and local ablative therapies are established therapeutic options (22, 23); in the intermediate stage, TACE is considered the standard treatment, while for advanced HCC, systemic therapy is the preferred treatment modality. As shown in the treatment-related MeSH term analysis, surgical treatments, such as liver resection and transplantation, were still shown to be the most common; however, they were overtaken by the rapid emergence of “Antineoplastic Agents.” The rise of “Antineoplastic Agents” appeared to be closely related to the increase of “Sorafenib.” Sorafenib is a multi-tyrosine kinase inhibitor and the first molecular targeted agent to show efficacy in advanced HCC (24), and it was included in one of the most common MeSH terms in our study. The overall survival of patients with HCC remains poor compared to other gastrointestinal, despite substantial research in systemic therapies; therefore, more effective systemic agents are needed.

Tumor cell lines are created by isolating cancer cells from a tumor and growing them in a laboratory setting to study the characteristics of the cancer cells, including their growth patterns, gene expression, and response to drugs (25–27). Hep G2 cells are one of the most used liver cancer cell lines due to their availability, easy handling, and the fact that they are derived from human HCC (28, 29). In our study, “Hep G2 Cells” among tumor cell lines were included in the top 10 basic research-related MeSH terms. According to our study, “Hep G2 cells’’ began to appear in HCC research after 2000 along with “MircroRNAs’’ and it subsequently showed a rapid increase in appearance. Researchers can use Hep G2 cells to manipulate the expression of specific microRNAs, which will enable them to study the potential of microRNAs as diagnostic and therapeutic targets for HCC (30–33).

Basic research on HCC is crucial for understanding the fundamental mechanisms of HCC and can lead to identifying potential therapeutic targets. Our LDA analysis showed that half of the entire number of studies on HCC focused on basic research. However, as found in other bibliometric studies, there has been little correlation between basic research and clinical studies in the research field of HCC (34, 35). One potential reason for this lack of correlation may have been a communication gap between the basic researchers and the clinicians. Another factor may have been the difficulty of translating basic research findings into clinical applications. Therefore, bridging these communication gaps through interdisciplinary collaboration and information sharing may help translate basic research findings into clinical research. Improving communication and collaboration between researchers and clinicians and investing in translational research efforts may also accelerate the progress toward more effective treatments for patients with HCC.

A bibliometric analysis may provide a comprehensive overview of a specific research field for performing quantitative analyses of academic literature. To the best of our knowledge, this is the first bibliometric analysis of HCC using machine learning techniques. Therefore, this study has important significance because it shows the developmental process of HCC research and possible future research directions.

However, our study had some limitations. First, only the Pubmed database was used in this study, and other databases, such as Web of Science, Scopus, and Embase, were excluded, which may have resulted in the exclusion of some research on HCC. Second, because only the MeSH term was used in the publication search, some publications not related to the subject may not have been included. This may have led to selection bias in our study. Finally, the newest research area of significance, such as immunotherapy, may not have been well discussed in this study due to the methodological problems inherent in bibliometric analyses and the low number of published studies in this area. However, we believe that machine learning bibliometric analysis may be a new tool that can enable scientists to comprehensively analyze large amounts of data.

In conclusion, this study was the first bibliometric study to analyze more than 60,000 publications on HCC over the past 30 years using machine learning. Our study showed that a machine learning bibliometric analysis may help researchers investigate large datasets, understand current trends, and identify research directions. Despite significant efforts in basic research, the connection with clinical practice is still lacking. Therefore, more efforts are needed to convert and apply the results of basic research to clinical treatment. Additionally, it was found that microRNAs have potential as diagnostic and therapeutic targets for HCC.

## Author contributions

Conceptualization, JH and S-WK; methodology, KL; formal analysis, KL; Writing-original draft preparation, HS and SS; Writing-review and editing, JH. All authors contributed to the article and approved the submitted version.
