# Supplementary material for: A systematic review of progress on hepatocellular carcinoma research over the past 30 years: a machine-learning-based bibliometric analysis
Source: Front Oncol. 2023 Aug 17;13:1227991. doi: 10.3389/fonc.2023.1227991 (PMC10471147; doi:10.3389/fonc.2023.1227991)
Supplement: Supplementary file 1 [file DataSheet_1.docx]

Supplementary Material

**A systematic review of progress on hepatocellular carcinoma research over the past 30 years: a machine-learning-based bibliometric analysis**

**Kiseong Lee^1^, Ji Woong Hwang^2^, Hee Ju Sohn^2^, Sanggyun Suh^2^, Sun-Whe Kim^2^**

*** Correspondence:** Ji Woong Hwang, MD, PhD: dattoree@gmail.com

# Supplementary Figure


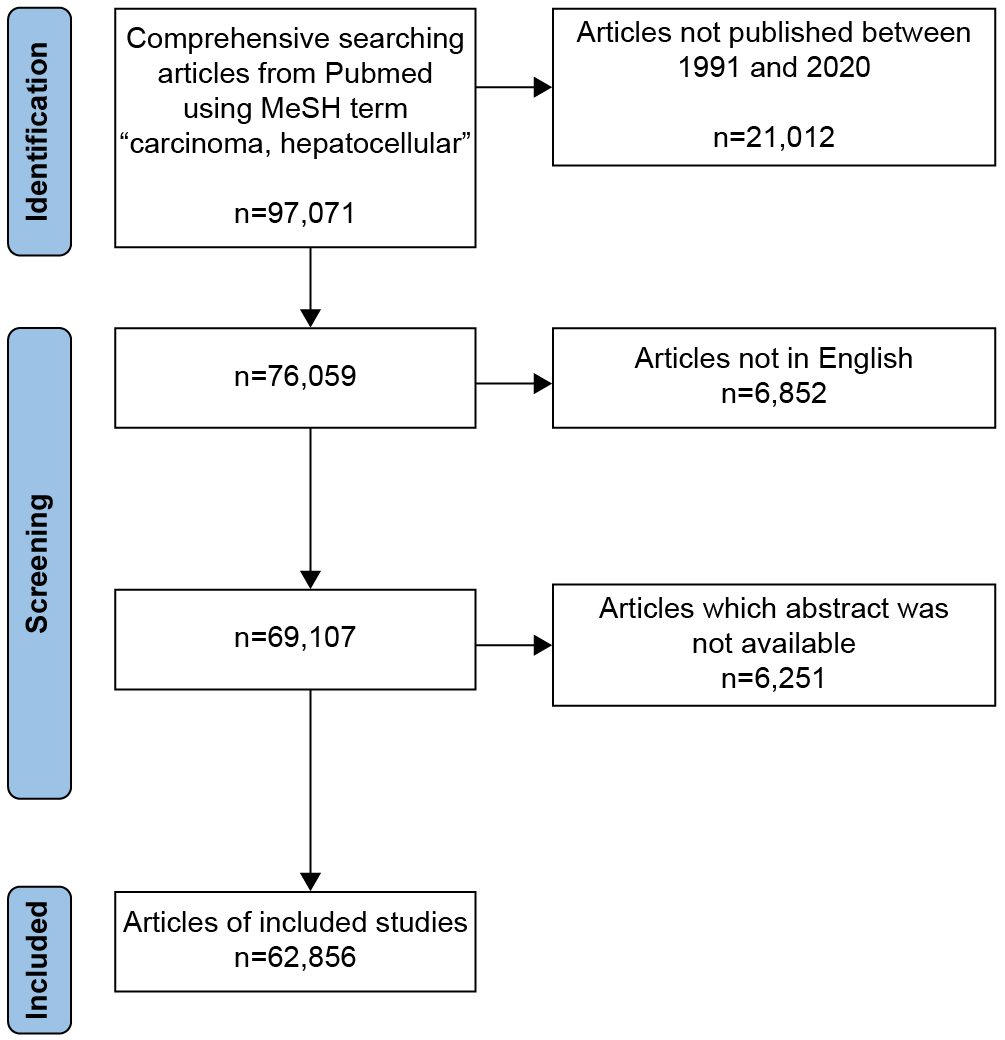


Figure S1. Flow diagram showing the selection of articles on hepatocellular carcinoma.

# Supplementary Tables

Table S1. Top 10 corresponding author’s country with the highest scientific production

|  | Country | Articles | Freq | SCP | MCP | MCP_Ratio |
| --- | --- | --- | --- | --- | --- | --- |
| 1 | China | 18,476 | 0.3507 | 16,998 | 1,478 | 0.08 |
| 2 | Japan | 9,248 | 0.1756 | 9,085 | 163 | 0.0176 |
| 3 | United States | 6,283 | 0.1193 | 5,720 | 563 | 0.0896 |
| 4 | South Korea | 3,625 | 0.0688 | 3,462 | 163 | 0.045 |
| 5 | Italy | 2,545 | 0.0483 | 2,312 | 233 | 0.0916 |
| 6 | Germany | 1,962 | 0.0372 | 1,745 | 217 | 0.1106 |
| 7 | France | 1,793 | 0.034 | 1,598 | 195 | 0.1088 |
| 8 | Spain | 834 | 0.0158 | 748 | 86 | 0.1031 |
| 9 | Egypt | 629 | 0.0119 | 506 | 123 | 0.1955 |
| 10 | Canada | 625 | 0.0119 | 529 | 96 | 0.1536 |

SCP, single country publications; MCP, multiple country publications.

Table S2. Most widely studied top 20 MeSH terms in hepatocellular carcinoma

| MeSH terms | Number of indicated MeSH terms/years | | | | | | |
| --- | --- | --- | --- | --- | --- | --- | --- |
|  | 1991–1995 | 1996–2000 | 2001–2005 | 2006–2010 | 2011–2015 | 2016–2020 | Total appearance |
| Carcinoma, Hepatocellular | 4,307 | 5,402 | 8,168 | 10,675 | 18,591 | 24,153 | 71,296 |
| Humans | 4,014 | 5,038 | 7,525 | 10,042 | 17,770 | 23,282 | 67,671 |
| Liver Neoplasms | 3,984 | 4,702 | 7,092 | 9,490 | 17,808 | 23,511 | 66,587 |
| Male | 2,058 | 2,797 | 3,975 | 5,371 | 10,546 | 12,797 | 37,544 |
| Female | 1,756 | 2,356 | 3,400 | 4,579 | 9,310 | 11,383 | 32,784 |
| Middle Aged | 1,621 | 2,269 | 3,133 | 4,249 | 7,883 | 9,959 | 29,114 |
| Aged | 1,203 | 1861 | 2,613 | 3,404 | 6,148 | 7,245 | 22,474 |
| Adult | 1,120 | 1,473 | 2,054 | 2,796 | 4,894 | 5,604 | 17,941 |
| Animals | 787 | 1,016 | 1,532 | 2,085 | 3,957 | 5,590 | 14,967 |
| Cell Line, Tumor | 0 | 3 | 959 | 2,445 | 3,711 | 4,911 | 12,029 |
| Prognosis | 201 | 358 | 664 | 1,008 | 2,581 | 4,028 | 8,840 |
| Retrospective Studies | 217 | 360 | 629 | 1,072 | 2,480 | 4,010 | 8,768 |
| Mice | 244 | 401 | 763 | 1249 | 2,551 | 3,487 | 8,695 |
| Treatment Outcome | 79 | 280 | 708 | 1,452 | 2,860 | 3,205 | 8,584 |
| Liver | 888 | 1,009 | 1,034 | 1,105 | 1,892 | 2,606 | 8,534 |
| Gene Expression Regulation, Neoplastic | 114 | 237 | 484 | 872 | 2,261 | 4,212 | 8,180 |
| Liver Cirrhosis | 618 | 762 | 999 | 1,208 | 1,822 | 2,353 | 7,762 |
| Cell Proliferation | 0 | 0 | 132 | 855 | 2,352 | 3,991 | 7,330 |
| Aged, 80 and over | 249 | 415 | 693 | 1,181 | 2,165 | 2,462 | 7,165 |
| Biomarkers, Tumor | 144 | 213 | 406 | 743 | 1,890 | 2,830 | 6,226 |
